# Supplementary material for: Exploring unsupervised feature extraction of IMU-based gait data in stroke rehabilitation using a variational autoencoder
Source: PLoS One. 2024 Oct 4;19(10):e0304558. doi: 10.1371/journal.pone.0304558 (PMC11452054; doi:10.1371/journal.pone.0304558)
Supplement: S1 Appendix — (DOCX) [file pone.0304558.s001.docx]

S1. Variational AutoEncoder settings evaluation


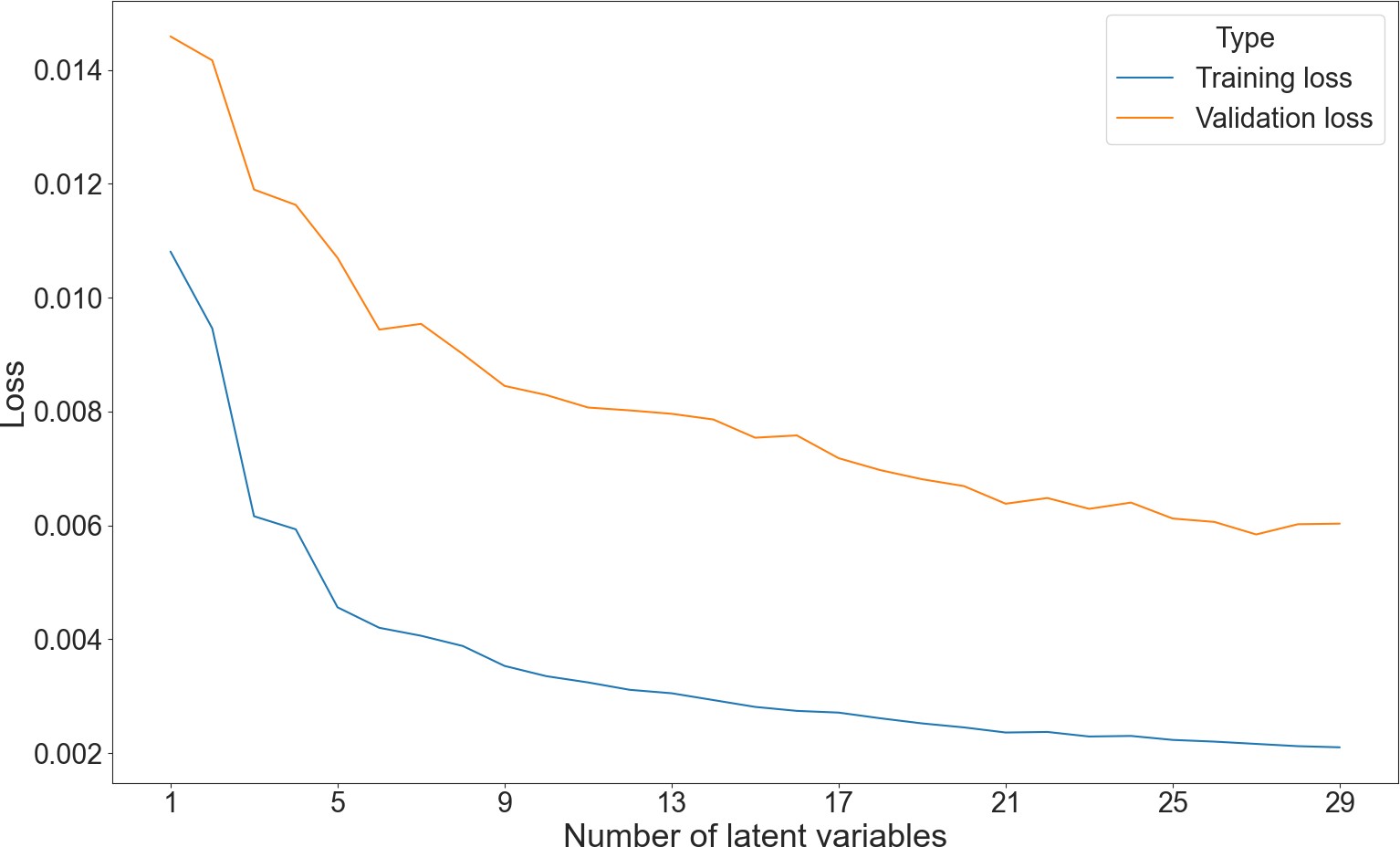


**Figure S1:** The loss function on both the training and test datasets for different numbers of latent features. To evaluate the model’s performance, the loss function was used, which combines the reconstruction error and the Kullback-Leibler divergence. The loss was calculated using the same set of train-test data for each number of latent features (N). This allowed for a comprehensive assessment of the loss across varying levels of feature representation. The code used to create the VAE is available at: <https://github.com/RichardFel/VAE>.
