# Supplementary material for: Exploring unsupervised feature extraction of IMU-based gait data in stroke rehabilitation using a variational autoencoder
Source: PLoS One. 2024 Oct 4;19(10):e0304558. doi: 10.1371/journal.pone.0304558 (PMC11452054; doi:10.1371/journal.pone.0304558)
Supplement: S2 Appendix — (DOCX) [file pone.0304558.s002.docx]

# **S2. Correlations**

**Table S1. Correlation between latent feature scores and gait speed.**

|  | L0*# | L1*# | L2* | L3 | L4 | L5*# | L6# | L7*# | L8# | L9 | L10 | L11# | Gait speed*# |
| --- | --- | --- | --- | --- | --- | --- | --- | --- | --- | --- | --- | --- | --- |
| L0*# | 1 |  |  |  |  |  |  |  |  |  |  |  |  |
| L1*# | -0,14 | 1 |  |  |  |  |  |  |  |  |  |  |  |
| L2* | 0,2 | 0,01 | 1 |  |  |  |  |  |  |  |  |  |  |
| L3 | 0,13 | -0,03 | 0,03 | 1 |  |  |  |  |  |  |  |  |  |
| L4 | 0,08 | -0,08 | -0,08 | 0,1 | 1 |  |  |  |  |  |  |  |  |
| L5*# | -0,22 | 0,14 | 0,02 | -0,02 | -0,02 | 1 |  |  |  |  |  |  |  |
| L6# | 0,02 | 0,08 | 0,1 | -0,02 | -0,1 | 0 | 1 |  |  |  |  |  |  |
| L7*# | -0,19 | 0,11 | -0,01 | -0,04 | -0,04 | 0,1 | -0,02 | 1 |  |  |  |  |  |
| L8# | -0,01 | 0,08 | 0 | -0,04 | -0,07 | -0,01 | -0,05 | 0,02 | 1 |  |  |  |  |
| L9 | -0,05 | 0,04 | 0,04 | 0,01 | 0 | 0,01 | 0,03 | 0,01 | -0,01 | 1 |  |  |  |
| L10 | 0,07 | -0,02 | 0,02 | 0 | -0,01 | -0,02 | 0,03 | -0,03 | 0,02 | -0,05 | 1 |  |  |
| L11# | 0,18 | -0,08 | 0,08 | 0,1 | 0,06 | -0,08 | 0,03 | -0,09 | 0,04 | 0,03 | 0,02 | 1 |  |
| Gait speed*# | 0,39 | -0,5 | -0,05 | -0,03 | -0,01 | -0,13 | -0,28 | 0,09 | 0,27 | 0,07 | 0 | 0,1 | 1 |
| The correlation coefficient was calculated using the pearson’s correlation coefficient. Correlations higher than 0.7 are marked in bold.  Variables with a good-excellent test-retest reliability are marked with a *. Variables which were significantly different between healthy controls and people after stroke are marked with a #. | | | | | | | | | | | | | |
