# Supplementary material for: Exploring unsupervised feature extraction of IMU-based gait data in stroke rehabilitation using a variational autoencoder
Source: PLoS One. 2024 Oct 4;19(10):e0304558. doi: 10.1371/journal.pone.0304558 (PMC11452054; doi:10.1371/journal.pone.0304558)
Supplement: S3 Appendix — (DOCX) [file pone.0304558.s003.docx]

S3. Validity of the stride detection algorithm

The validity and reliability of the stride detection algorithm was evaluated prior to the current study. The validity was evaluated by measuring gait of healthy participants with three IMUs (2 at the feet and 1 at the lower back) and comparing the outcomes to a golden standard. In validation assessment, we intended to imitate various gait patterns via three walking paths in which foot placements were indicated. Participants were instructed to walk symmetric or asymmetric at normal and slow speed.

*Protocol*

In total 8 healthy participants walked a total of three to six times on three different walking paths. Markings with ascending numbers were drawn on the ground to indicate where the participants should place their feet during the assessment. The markings were placed at a distance 20 cm, 40 cm and 60 cm. Additionally, participants were instructed to walk symmetric or asymmetric at a low frequency (1 step every 2 seconds) or high frequency (1 step every second). The walking paths are illustrated in figure A1.

*Data processing*

To determine the strides in during the 2MWT, a custom-made stride detection algorithm was applied. First, the average time per stride was estimated based on the dominant frequency found in the medio-lateral acceleration using a Fast Fourier Transform. Second a peak detection algorithm was used to identify foot contact in the vertical acceleration. A false negative and false positive peak detection was used with the assumptions that 1) the stance phase between peaks should be of a certain minimum and maximum; 2) a peak was of above a certain threshold. Both assumptions depended on the individual’s their gait.

*Validity*

In total 37 measurements were collected. After the assessment the number of strides were counted based on the number in the marking on which the participants ended plus the number of completed rounds times the markings in the walking path. The covered distance was calculated by multiplying the number of strides with the distance between strides in the walking path. The number of strides and total distance were considered the golden standard and compared to the outcomes of the custom-made stride detection algorithm. Nine measurements were excluded from analysis, six because of missing data and three because of faulty measurements. The results are reported in table B1. Based on the results we concluded that there are no remarkable differences between the step detection algorithm and the golden standard.


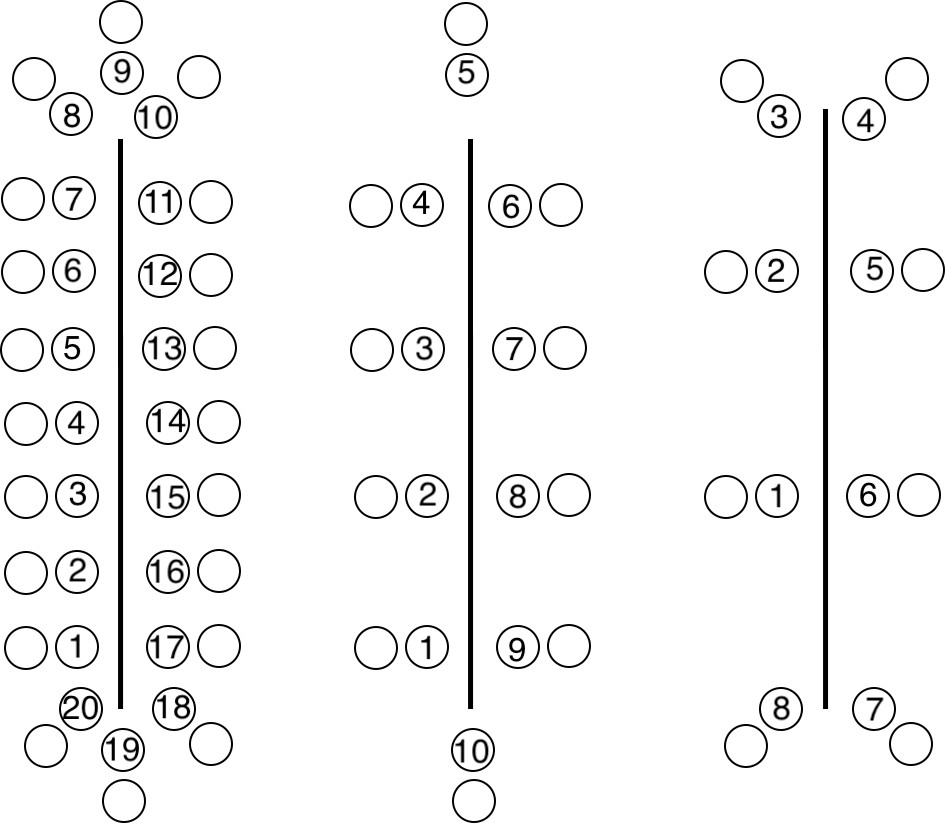


**Figure S2.** Participants walked on three 14-meter walking paths with numbered markings at 20 cm, 40 cm and 60 cm. Participants took right turns. The three inertial measurement units were placed at the low back and on top of the left and right foot. Elastic bands were used to hold the sensors in place. The depicted walking paths have less markings than the walking paths used for testing.

**Table S2**: Comparison between the outcomes of the golden standard and stride detection algorithm

|  | Measurement | Mean (SD) [min, max] | Pearson’s r | Root Mean Square Error | Absolute average difference |
| --- | --- | --- | --- | --- | --- |
| Strides left foot | GS | 49.3 (15.5) [27,88] | r(28)=0.97, p<0.01 | 3.90 | 1.96 |
|  | SDA | 48.4 (16.1) [24, 88] |  |  |  |
| Strides right foot | GS | 49.3 (15.5) [27,88] | r(28)=0.98, p<0.01 | 3.36 | 1.60 |
|  | SDA | 47.8 (16.2) [23, 88] |  |  |  |
| Steps low back | GS | 98.5 (31.0) [53, 176] | r(28)=0.98, p<0.01 | 6.51 | 2.46 |
|  | SDA | 97.2 (32.7) [47, 178] |  |  |  |
| Distance left foot | GS | 29.9 (22,6) [7.6, 105.6] | r(28)=0.97, p<0.01 | 5.93 | 4.11 |
|  | SDA | 30.9 (21.0) [8.1, 97.8] |  |  |  |
| Distance right foot | GS | 29.9 (22.6) [7.6, 105.6] | r(28)=0.97, p<0.01 | 5.69 | 3.90 |
|  | SDA | 32.3 (22.1 [8.1, 109.6] |  |  |  |

Abbreviations: GS = Golden standard; SDA = Step Detection Algorithm; SD = Standard deviation; Min = Minimum; Max = Maximum.
