# Supplementary material for: Exploring unsupervised feature extraction of IMU-based gait data in stroke rehabilitation using a variational autoencoder
Source: PLoS One. 2024 Oct 4;19(10):e0304558. doi: 10.1371/journal.pone.0304558 (PMC11452054; doi:10.1371/journal.pone.0304558)
Supplement: Attachment — Submitted filename: Point-by-point reply.docx [file pone.0304558.s004.docx]

**Point-by-point reply**

We want to thank the editor and the reviewers for their valuable time and insightful feedback. This has helped us to improve the manuscript. In the revised manuscript, we have marked all modifications in red. Below, you will find our responses to each of the comments and questions, organized by number. Each response directly follows the related comment or question. We have also included the sections of text that were copied, altered, and supplemented in response to your feedback below each respective comment or question.

Editor #1

*1. Please ensure that your manuscript meets PLOS ONE's style requirements, including those for file naming. The PLOS ONE style templates can be found at*

1. Answer:
We have adjusted the manuscript so that it meets the requirements.

*2. Please note that PLOS ONE has specific guidelines on code sharing for submissions in which author-generated code underpins the findings in the manuscript. In these cases, all author-generated code must be made available without restrictions upon publication of the work. Please review our guidelines at https://journals.plos.org/plosone/s/materials-and-software-sharing#loc-sharing-code and ensure that your code is shared in a way that follows best practice and facilitates reproducibility and reuse.*

2. Answer:
A license has been added to the code, and the code description has been improved. The code is now available on Zenodo with the DOI: 10.5281/zenodo.10878639.
The link to the software and tool has been moved to the data availability statement.

*3. Thank you for stating the following financial disclosure:*

*“This study is independent research and was funded by: SIA-RAAK (RAAK.PRO.03.006). SMB was funded by a VIDI grant (016.Vidi.178.014) from the Dutch Organization for Scientific Research (NWO).”*

*Please state what role the funders took in the study. If the funders had no role, please state: “The funders had no role in study design, data collection and analysis, decision to publish, or preparation of the manuscript.”*

3. Answer:

We have added the following sentence to the manuscript: “The funders had no role in study design, data collection and analysis, decision to publish, or preparation of the manuscript.”

*4. Your ethics statement should only appear in the Methods section of your manuscript. If your ethics statement is written in any section besides the Methods, please delete it from any other section.*
4. Answer:
We have removed the ethics statement from the declarations section.

*5. We note that you have indicated that there are restrictions to data sharing for this study. PLOS only allows data to be available upon request if there are legal or ethical restrictions on sharing data publicly. For more information on unacceptable data access restrictions, please see <http://journals>.plos.org/plosone/s/data-availability#loc-unacceptable-data-access-restrictions.*

*Before we proceed with your manuscript, please address the following prompts:*

*a) If there are ethical or legal restrictions on sharing a de-identified data set, please explain them in detail (e.g., data contain potentially identifying or sensitive patient information, data are owned by a third-party organization, etc.) and who has imposed them (e.g., a Research Ethics Committee or Institutional Review Board, etc.). Please also provide contact information for a data access committee, ethics committee, or other institutional body to which data requests may be sent.*

5. Answer:

We are working on a repository to make the data used in this paper available. We expect to complete this step somewhere in the next two months. Until the de-identified data set is available, the data is available on request via the corresponding author. We have added this information to the data availability section.

Reviewer #1

*1. The title would benefit from including the main finding of the study; for example, can the variational encoder represent movement features well enough?*
1. Answer:
Thank you for your suggestion. However, we wanted to emphasize the explorative nature of the study as it is one of the first in this field. Moreover, there is not one main finding or conclusion, since we evaluated both the effectiveness of the dimensionality reduction using a VAE and the psychometric properties of these latent features scores. Therefore, we have decided to keep the title as is.

*2. (31-33) Is this the problem addressed? If so, it may help addressing my previous comment about the title. Also, what follows in lines 34-36 is not very clear to me, at least without reading the rest of the manuscript. It looks like you are attempting a classification task here (contrary to the unsupervised approach mentioned earlier and also in the title)? Or you are checking whether there is clustering that could be done and that can discriminate the healthy and pathological group? I suggest rephrasing and making it clearer. Similarly, it would be advisable to better specify what test-retest refers to (different sessions? split in dataset from same session?)*
2. Answer:
The main problem addressed here is that there are countless ways to obtain information from complex time-series data, making it difficult to determine which characteristics are the most relevant. Therefore, we explored a different method of extracting relevant information for IMU-data using an unsupervised method and evaluated the psychometric properties of this new method.
We were not attempting to make a classification based on the data. The VAE is used to first reduce the IMU-data to a few latent features, the properties of which are then further explored.
We have rephrased the abstract (see textual changes below) to improve its readability. However, we were limited to the maximum word count, which affected the amount of information that we could share in the abstract.
2. Textual change (lines 108-122):
“Variational AutoEncoders (VAE) might be utilized to extract relevant information from an IMU-based gait measurement by reducing the sensor data to a low-dimensional representation. The present study explored whether VAEs can reduce IMU-based gait data of people after stroke into a few latent features with minimal reconstruction error. Additionally, we evaluated the psychometric properties of the latent features in comparison to gait speed, by assessing 1) their reliability; 2) the difference in scores between people after stroke and healthy controls; and 3) their responsiveness during rehabilitation.

Methods
We collected test-retest and longitudinal two-minute walk-test data using an IMU from people after stroke in clinical rehabilitation, as well as from a healthy control group. IMU data were segmented into 5-second epochs, which were reduced to 12 latent-feature scores using a VAE. The between-day test-retest reliability of the latent features was assessed using ICC-scores. The differences between the healthy and the stroke group were examined using an independent t-test. Lastly, the responsiveness was determined as the number of individuals who significantly changed during rehabilitation.“

*3. (99) The introduction reads well and has a nice flow, leading the reader through the problem under analysis and the broader scope of the work carried out. However, when it comes to the statement of aim and objectives, I suggest more information is provided and more attention is paid to describing the items under investigation. For example, an overview of AE and perhaps most importantly of the distinguishing features of VAE (as opposed to AE) should be provided, as well as a brief discussion about why this architecture has been chosen over others.
Also, more detail would be beneficial re: (1) what type of reliability, (2) what reference for assessing reconstruction capability (is it on original timeseries?), and (3) the nature of the analysis of group (pathological/control) features and monitoring (see comment for Abstract).*
3. Answer:
Thank you for your compliment and recommendation. We have extended the introduction to include information about why a Variational Autoencoder was chosen instead of a regular Autoencoder.
Regarding your comment about the model architecture. The goal of this study was to explore the applicability of a deep-learning algorithm on IMU-based gait data, less so to determine the best or optimal model to achieve this goal.
We chose for a VAE as an unsupervised data-reduction method as it has been used in previous research with time-series data. We think that future studies should try to evaluate different models and model architectures. We have added this as a recommendation in the discussion section.
3. Textual change (lines 223-239; 247-253; 815-820):
“An alternative approach to obtain features from time-series data that requires fewer theoretical assumptions is the utilization of data-driven methods, which can reduce data to a pre-defined number of latent features to describe the data. AutoEncoders (AE) are an example of such algorithms [13]. An AE is a model that consists of an encoder, a latent layer with latent features, and a decoder. The encoder reduces the dimensionality of the input data to a set number of latent features in the latent layer. Subsequently, the decoder tries to reconstruct the input data given the latent-feature scores. The AE learns by minimizing the difference between the input and reconstructed data, forcing it to learn a compact, low-dimensional representation of the data. AEs share similarities with principal component analysis (PCA), however, they are capable of modelling non-linear functions as well. The downside of the AE is that it does not constrain the distribution of the latent features, making them unsuitable to generate new data, less robust to input noise, and less reliably with new unseen data. Variational AutoEncoders (VAE) address the regularization issues of the AE by forcing the latent-feature scores to be normally distributed via an extension of the loss function. In this new loss function, the differences between the distribution of the latent-feature scores and a standard Gaussian distribution are evaluated as well as the differences between the input and reconstructed signal. “

“The present study aimed to explore if a VAE can be applied to extract a reduced set of latent features from an IMU-based measurement of gait in clinical stroke rehabilitation while maintaining high accuracy in signal reconstruction. Moreover, we aimed to investigate the relevance of the latent features by evaluating the psychometric properties of the latent-feature scores in comparison to gait speed by determining 1) the between-day test-retest reliability of the latent-feature scores; 2) the differences in latent-feature scores between people after stroke and healthy controls; 3) and if the latent features are responsive to changes during rehabilitation. “

“Lastly, in this study, a VAE was used to extract relevant information from raw IMU-data during gait. Since this study was exploratory, no parameter optimization was applied to achieve optimal model outcomes. Furthermore, there are different types of machine-learning methods, such as t-SNE, that could effectively reduce IMU-data. Future research is required to identify the best type of model and the optimal model architecture to obtain relevant information IMU-based gait data.”

*4. (110-111) Ditto. Is test-retest from 2 different sessions? How much time apart?*
4. Answer:
The retest was administered the subsequent day. We have changed the text to between-day test-retest measurements.
4. Textual change (lines 272-275):
“The dataset included both between-day test-retest measurements and longitudinal data. Additionally, between-day test-retest data were collected from a control group, including adults, and elderly participants at a nursing home. The retest data was measured the subsequent day at approximately the same time of the day.”

*5. (125) Were 8gs enough to avoid clipping/saturation? Also, what was the rationale behind sensor location? Was it arbitrary, driven by specific reasons (practicality?) or informed by previous work, literature etc.? I appreciate there is no perfect choice and not much knowledge yet about best wearable locations… however it may be beneficial for the reader/other scientist attempting similar approached to know what the thought process was, leading to that selection.*
5. Answer:
Prior to this study, we extensively tested the different sensor settings and locations. We found that 8G and 500°/s were sufficient to avoid clipping while walking in healthy controls and the stroke population.
Moreover, we chose to place the sensors on the feet, which is a common in research to calculate spatio-temporal parameters, such as gait speed, and because it was the most feasible for our population. We have added a reference to the text.

5. Textual change (lines 284-285):
“Data were collected using two unsynchronized Inertial Measurement Units (IMUs) positioned on the left and right foot [19].”

*6. (127) I guess it may not matter much anyway (given the scope of your work and the sample analysed), but I think it may be good to know how many samples of aided/unaided gaits from the same participant were collected (as % of the total). There may be an argument that the features of walking are different, but some characteristic features are carried over for the same individual.*
6. Answer:
Around 10% of the measurements were from individuals that walked with and without a walking aid. We have added this information to the results section. Characteristics might indeed be similar within one individual. However, gait with and without a walking aid in an individual after stroke can also be very different as the walking aid offers a considerable amount of stability often resulting in an increase in walking speed. The similarity in features within an individual was also mentioned as a limitation in the discussion.

6. Textual change (lines 538-539):
“Thirty-two (10.5%) two-minute walk test measurements from the same individual measured with and without walking aid at one time point.”

*7. (129) What assessment? Do you mean protocol? In any case, please include a summary here, as readers should not need to access another document to have they key information.*
7. Answer:
You are correct, it is undesirable to require reading a previous study. We have removed this sentence altogether to avoid confusion, as there is limited extra information available in the other study about the specific protocol.

*8. (139) Was 104 Hz imposed by the system used? Does it depend on the no. of units used?*
8. Answer:
Yes, the maximum sampling frequency of the used sensor was 104 Hz. Previous work indicated that this frequency was sufficient to reliably measure gait features in people after stroke during rehabilitation. Moreover, we expect the most relevant frequencies to be below 50 Hz (Nyquist Frequency)

*9. (141-151) Please provide the key aspects of the step-detection algorithm, and its validation outcomes. What is the role of gait event detection in the separation of data? Were epochs 5.12 s long (and therefore included multiple steps/strides?) What is the scope of overlapping and why 50%? Why did not you scale to Z-scores directly? Overall a figure to give a visual description of the steps followed would be beneficial.*
9. Answer:
We have added information regarding the validity of the step detection to the appendix.
We indeed included sections of 5.12 seconds to include multiple strides in the data, as a shorter duration might be too short to capture relevant information and a longer duration makes it harder for a model to reconstruct the data. We applied a 50% overlap to increase the available data for model training, since a VAE requires a considerable amount of data to train the model.
We used the z-scores on the mean and standard deviation to detect outliers. It is unclear how we would convert the processed data of sample size 512 x 6 to z-scores to perform a different type of outlier detection.
Lastly, we have adjusted figure 1 to make it clearer how the data was processed, and which data were included in the model.

9. Textual change (lines 320-336; Supplements S3; 371-392):
“Third, a step detection algorithm was applied to identify the foot contacts and the stance phases within the gait measurement [9]. Fourth, spanning from the second to second-to-last stride, all 2MWT were split up into epochs of 512 samples, starting in a stance phase, with approximately 50% overlap. Data were split up into segments with 50% overlap to increase the amount of data for the VAE to learn, as a considerable amount of data is required to accurately estimate the model coefficients. An epoch length of 512 samples (5.12 seconds) was chosen to encompass multiple strides per epoch, thus capturing relevant information about an individual’s gait pattern. “

“Appendix S3. Validity of the stride detection algorithm

The validity and reliability of the stride detection algorithm was evaluated by measuring gait of healthy participants with three IMUs (2 at the feet and 1 at the lower back) and comparing the outcomes to a golden standard. In the validation assessment, we intended to imitate various gait patterns via three walking paths in which foot placements were indicated. Participants were instructed to walk symmetric or asymmetric at normal and slow speed.

Appendix B.1 protocol

In total 8 healthy participants walked a total of three to six times on three different walking paths. Markings with ascending numbers were drawn on the ground to indicate where the participants should place their feet during the assessment. The markings were placed at distances of 20 cm, 40 cm and 60 cm. Additionally, participants were instructed to walk symmetric or asymmetric at a low frequency (1 step every 2 seconds) or high frequency (1 step every second). The walking paths are illustrated in figure A1.

Appendix B.2 Data processing

To determine the strides in during the 2MWT, a custom-made stride detection algorithm was applied. A cutom-made algorithm was used because existing stride-detection algorithms, such as continuous wavelet transform and template matching were inadequate for accurately detecting strides in very slow, poor, or asymmetric gait. To detect the strides, firstly, the average time per stride was estimated based on the dominant frequency found in the medio-lateral acceleration, using the Fast Fourier Transform. Secondly, a peak-detection algorithm was used to identify foot-contact in the vertical acceleration. Subsequently, a false-negative peak detection was applied to minimize errors by comparing the sample difference between peaks to the expected between-peak difference (average time per stride). In the negative peak detection, the between-peak sample difference was evaluated; if the difference exceeded more than 1.5 times the expected difference, then the previously described peak detection was applied with 0.75 * the minimal peak height. After the false-negative peak detection, the stance phases were determined based on the stationary periods between foot-touches (peaks), where a stationary period was defined minimally as 0.2 consecutive seconds below a gyroscope- and acceleration-threshold (mean acceleration magnitude + standard deviation acceleration magnitude and mean gyroscope magnitude). In case no stance phase between foot-touches could be identified, a false-positive peak detection was applied to remove the lowest peak before determination of the stance phase. Swing phases were defined as the periods between stance phases. To compute spatial gait features, the accelerometer and gyroscope data were aligned with the vertical (VT)(upward: positive), medial--lateral (ML) (right: positive), and anterior--posterior (AP) axes (anterior: positive), and corrected for the effects of gravity using a sensorfusion algorithm. Lastly, a zero-velocity update (ZUPT) was applied to correct for gravity prior to the calculation of spatial gait features.

Appendix B.3 Validity

In total 37 measurements were collected. After the assessment the number of strides were counted based on the number in the marking on which the participants ended plus the number of completed rounds times the markings in the walking path. The covered distance was calculated by multiplying the number of strides with the distance between strides in the walking path. The number of strides and total distance were considered the golden standard and compared to the outcomes of the custom-made stride detection algorithm. Nine measurements were excluded from analysis, six because of missing data and three because of faulty measurements. The results are reported in table B1. Based on the results we concluded that there are no remarkable differences between the step detection algorithm and the golden standard.

Figure B1. Participants walked on three 14-meter walking paths with numbered markings at 20 cm, 40 cm and 60 cm. Participants took right turns. The three inertial measurement units were placed at the low back and on top of the left and right foot. Elastic bands were used to hold the sensors in place. The depicted walking paths have less markings than the walking paths used for testing.

Table B1: Comparison between the outcomes of the golden standard and stride detection algorithm

|  | Measurement | Mean (SD) [min, max] | Pearson’s r | Root Mean Square Error | Absolute average difference |
| --- | --- | --- | --- | --- | --- |
| Strides left foot | GS | 49.3 (15.5) [27,88] | r(28)=0.97, p<0.01 | 3.90 | 1.96 |
|  | SDA | 48.4 (16.1) [24, 88] |  |  |  |
| Strides right foot | GS | 49.3 (15.5) [27,88] | r(28)=0.98, p<0.01 | 3.36 | 1.60 |
|  | SDA | 47.8 (16.2) [23, 88] |  |  |  |
| Steps low back | GS | 98.5 (31.0) [53, 176] | r(28)=0.98, p<0.01 | 6.51 | 2.46 |
|  | SDA | 97.2 (32.7) [47, 178] |  |  |  |
| Distance left foot | GS | 29.9 (22,6) [7.6, 105.6] | r(28)=0.97, p<0.01 | 5.93 | 4.11 |
|  | SDA | 30.9 (21.0) [8.1, 97.8] |  |  |  |
| Distance right foot | GS | 29.9 (22.6) [7.6, 105.6] | r(28)=0.97, p<0.01 | 5.69 | 3.90 |
|  | SDA | 32.3 (22.1 [8.1, 109.6] |  |  |  |

Abbreviations: GS = Golden standard; SDA = Step Detection Algorithm; SD = Standard deviation; Min = Minimum; Max = Maximum. “

“Fig 1: The hierarchy of the data and the VAE used in this study. Data were processed and split up into epochs of 512 samples with 6 dimensions (triaxial acceleration and angular velocity). The encoder (green) and the decoder (blue) consisted of 3 mirrored convolutional layers with a size of 256, 128 and 64 nodes. These layers were configured with 32, 64, and 128 filters, respectively, and employed a kernel size of 3. The activation function used throughout the model was a hyperbolic tangent. The latent layer contained 12 normally distributed latent features. The model was trained by comparing the input to the reconstructed output. An Adam optimizer with a learning rate of 0.001 was used. The loss function consisted of two aspects: 1) the difference between the original and reconstructed signal; 2) the difference between the distribution of the latent features and a Gaussian distribution. The VAE was created in Python using TensorFlow version 2.11.0 and is available via: <https://zenodo.org/doi/10.5281/zenodo.10878458> [29].
* The actual sensor data consisted of a two-minute measurement with six dimensions. For demonstration purposes a one-dimensional signal was visualized for ten seconds.”

*10. (163) Please describe the matrix more clearly. I guess the 6 layers are the 3D accelerations and 3D angular velocities? It is still unclear to me what data goes into the 512 columns, are those all datapoints from the same person? Figure 1 does not help in this sense, and probably should be revised to improve clarity about data input. Similarly, more information should be provided about the selection of the model architecture, and whether any hyperparameter optimisation has been carried out, for example to determine the best configuration of layers, number of nodes per layer, dropout, activation function and, perhaps more importantly, number of latent variables (why 12?). An argument (which I mentioned earlier) would be about the model used… one could ask why VAE? If any other test (other architectures or approaches) has been carried out, it would be good to mention it, so that the journey leading to what presented here is clear (which is informative per se).*
10. Answer:
Thank you for your suggestions. We have tried to improve the model description. It is important to note that the aim of this study was to explore a new method to process IMU-based data using a different method than the conventional way of data processing. Therefore, our intent was not to optimize the model parameters and thus we did not use hyperparameter optimization. Moreover, we used the VAE as it has been used in previous work on time-series data. A comparison between different models is outside the scope of this study.

We chose 12 features as this seemed like a reasonable number to capture the most important features of the data, while allowing for further exploration of psychometric properties. An argument can be made that more latent features would result in a better model outcome, however, this would also increase the risk of overfitting and makes subsequent analysis less concise. Future research should further explore the different settings and options to optimize the model results, which is also recommended in the discussion.
The revised version of the figure is given in the previous question.
10. Textual change (lines 363-372; 817-822):

“The input and output of the VAE used in this study consisted of a 512 X 6 matrix (an epoch), where 512 is the number of data points, and 6 the triaxial acceleration and angular velocity. The encoder and decoder both comprised three mirrored convolutional layers. The latent layer contained 12 latent features. In summary, the VAE learned by reducing an epoch (512X6) to 12 latent features, which are then used to reconstruct the original signal. The VAE tries to reduce the differences between the original and reconstructed signal, while forcing the latent features to be normally distributed. Additional details regarding the chosen number of latent features are provided in Appendix 1. Fig 1 provides a simplified visual representation of the VAE architecture. The source code of the project is available via: https://zenodo.org/doi/10.5281/zenodo.10878458. “

“Lastly, in this study, a VAE was used to extract relevant information from raw IMU-data during gait. Since this study was exploratory, no parameter optimization was applied to achieve optimal model outcomes. Furthermore, there are different types of deep-learning models, such as t-SNE, that could effectively reduce IMU-data. Future research is required to identify the best type of model and the optimal model architecture to obtain relevant information IMU-based gait data.”

*11. (187) Do you mean when there was divergence in performance between training and validation?*
11. Answer:
Yes, that is exactly what we meant to say. We have rephrased the sentence to make it clearer.
11. Textual change (lines 402-404):
“Early stopping was employed if there was no further improvement in performance to prevent overfitting.”

*12. (191) If I understand correctly, you are assuming that the differences in the healthy group reconstruction (i.e. real healthy – reconstructed healthy) generated from a model trained with the stroke data only are (or may be) the aspects distinguishing healthy from pathological gait? If so I think:
1) better info should be given since the start of the manuscript (see my previous comments, where I thought you were attempting a classification based on the latent features)
2) some discussion should be presented re: this assumption, as that difference may be the sum of the error from the reconstruction in general (i.e., how good the model is in representing a stroke patient gait) and of the difference between groups? Also, how variance within those two groups is taken into account?
If I have not understood well… then I apologise, but the description may need some change to improve clarity!*
12. Answer:
We trained the model indeed on stroke data to make a stroke specific model and used this to evaluate differences between healthy and stroke. Therefore, it is indeed possible that the differences we find might be explained by a higher reconstruction error of gait data of the healthy population (then the latent features would be less accurate). For this reason, we evaluated the between-day test-retest data, including both individuals after stroke and healthy participants. If there is a random error in the latent features scores of the healthy individuals we would expect the ICC-scores to be small (which was true for some latent features).
Nevertheless, we had incorporated this section to the limitations in the previous manuscript as it might be considered as a limitation of the methods.

12. Textual change (lines 796-803):
“Secondly, we evaluated the reliability and the difference between the healthy and stroke groups using the outcomes of the latent features, while we demonstrated that the reconstruction error of the model on healthy participants was considerably higher than the reconstruction error of data from people after stroke. Consequently, the latent feature scores for healthy controls might lack precision, potentially affecting both the reliability of these scores and the observed differences between healthy and stroke groups. However, if we assume that the reconstruction errors occur randomly, we are more likely to observe smaller differences between groups and lower reliability in our measurements.”

*13. (195-200) I do not think this part of the processing is clear enough… and unfortunately Figure 2 does not help much… I suggest rephrasing to improve clarity and revising Figure 2.*
13. Answer:
We have rephrased this section to make it clearer how we analyzed the data. We first created and evaluated the model on the data of the people after stroke and the healthy controls. Next, we averaged the latent scores per measurement and evaluated the psychometric properties of these averaged scores. Figure 2 was used to describe which data is used for which part of the analysis. We have changed the figure’s description to make this clearer.
13. Textual change (lines 411-453; 492-522):
“Next, the trained VAE was used to calculate the latent feature scores per epoch. Since one 2MWT measurement consisted of multiple epochs, the average value of the twelve latent features was calculated for both the left and the right foot, resulting in twelve averaged latent feature score per 2MWT. These averaged latent feature scores were used in further statistical analysis.

2.6 Statistical Analysis
The statistical analysis consisted of three parts. In the first part, the between-day test-retest reliability of the latent feature scores were calculated to indicate if the latent features are consistent. In the second part, the differences in latent feature scores between healthy and individuals after stroke were calculated to determine if the latent features capture information that differs between healthy and stroke. In the third part, the changes in the latent feature scores over time during rehabilitation were determined to indicate if the latent features are responsive to rehabilitation. Figure 2 is a visualization of the type of data that is used to for the creation and evaluation of the model, and to evaluate the reliability, differences and responsiveness. “

“Figure 2: Visualization of the type of data that is used for the specific research question. The dataset included both test-retest data from people after stroke (red) and healthy individuals (green), and longitudinal data from people after stroke (blue). The data from the people after stroke was used to train, test, and validate the VAE. The trained VAE was then used to evaluate the model fit, via the reconstruction error, on the data of the healthy control group. Next, the average value per latent feature was calculated for each measurement. These averaged latent features scores were used to 1) determine the between-day test-retest reliability using the test-retest data of the people after stroke and the healthy controls; 2) determine if people after stroke significantly changed during rehabilitation using the longitudinal data; and 3) evaluate the differences between the healthy control group and the stroke group.”

*14. (248-249) There is repeated info from methods here.*

14. Answer:
Thank you for your suggestion. We tried to avoid repetition as much as possible, while guiding the reader through the text. We have removed the following lines from the text:
This process was repeated ten times so that all people after stroke were once in the external validation set.

*15. (251) Are these errors in standardised scores? It would be useful to have an idea of the reconstruction errors in the original units as well… to have a better idea of what they could mean from a biomechanical perspective*
15. Answer:
The presented error scores were standardized scores. We have added the errors in units behind the standardized squared errors.
15. Textual change (lines 561-562):
“The non-standardized mean absolute error per epoch for the acceleration and angular velocity was 0.15G (±0.05) and 0.59º/s (±0.18).”

*16. (279) The reference to gait speed comes out a bit unexpected, as it was not mentioned earlier in methods.*
16. Answer:
We agree that it was not clear why we included gait speed and how this calculated.
We have added several references in the method section so that it is more in line with the presented results.
16. Textual change (lines 111-114; 311-321):
“Additionally, we evaluated the psychometric properties of the latent features in comparison to gait speed, by assessing 1) their reliability; 2) the difference in scores between people after stroke and healthy controls; and 3) their responsiveness during rehabilitation.”

The data processing to compute gait speed and prepare the data for the VAE is described below.

“Computation of gait speed was done in seven steps. First, data were downsampled from 104 Hz to 100 Hz. Second, the gyroscope data were corrected for the gyroscope offset derived from a static measurement. Third, a custom-made step-detection algorithm was applied to determine stance and swing phases in the 2MWT (Appendix S3). Fourth, a sensor-fusion algorithm was used to transform acceleration from a local to a global reference frame by combining the accelerometer and gyroscope data [38]. Fifth, the linear acceleration in the anterior-posterior direct was integrated once to determine the gait speed. Sixth, a Zero-Velocity Update was applied to set velocity to zero during stationary phases of walking, thereby reducing estimation errors [39]. Seventh, the corrected gait speed was integrated to determine position. This calculation enabled us to measure the total distance covered in the two-minute assessment, and thus, the gait speed. “

DISCUSSION
*17. (318) Similarly to what observed for the Results section, it would be better to discuss what is meant for “high accuracy” and its interpretation from a biomechanical perspective (for example, are those differences meaningful when reconstructed as original quantities? What best and worst case scenario, beyond average across gait cycle and multiple people/strides?)*
17. Answer:

We agree that a high accuracy might be too vague to interpret. Therefore, we have changed the wordings of this section in the discussion to small differences between the original and reconstructed signal. It is difficult to fully describe how large the error is beyond the mean absolute error and to uncover if these differences are meaningful (when is the difference too large?). Especially considering that the metrics used to describe the differences are an average, which raises a new question (how stable is the reconstruction over time; how good is the reconstruction in the different gait phases). We think that diving deeper into this problem is outside the scope of this explorative study. Nevertheless, we have added a section to the discussion to mention these uncertainties.
17. Textual change (lines 720-722, 762-772):
“We found that a VAE can effectively reduce a gait measurement to twelve latent features, as shown by the small differences between the original and reconstructed IMU data.”
“The differences between the original and reconstructed IMU-based gait data were evaluated using the reconstruction error, computed as the mean squared error for the standardized epoch and the average absolute error for both the acceleration and angular velocity. These measures are an indication of the model performance on the whole epoch, thus how well the VAE can reconstruct the data on average. However, in the gait data used, there might be some timeframes in the signal that are more relevant than others. For instance, samples around the foot placement might contain more relevant information that the same number of samples during the stance phase. With the current metrics, it is difficult to determine how well the VAE reconstructs these specific parts of the signal. Moreover, it is unclear if the reconstruction error remains stable over time, thus if the error has the same size during the first hundred samples as the last hundred samples. Future studies should take this into consideration when using a VAE.”

*18. (322) “higher…than speed” between what conditions or groups?*
18. Answer:
We have added information regarding the effect size to the text.
18. Textual change (lines 725-726):
“Interestingly, one latent feature demonstrated a higher effect size than gait speed in the difference between the healthy control group and individuals after stroke.”

*19. (327-331) These arguments may need some further explanations/examples to make them more accessible to the wider readership*
19. Answer:
We agree that this is an interesting point and have added some text to make it clearer.
19. Textual change (lines 732-738):
“This raises the question if the changes in gait, measured with the latent feature scores, are an indication of between-individual differences in recovery and compensation strategies, i.e. functional adaptations to overcome impairments, or simply due to measurement error [31, 32]. For instance, some individuals might reduce their gait asymmetry as a result of neuroplasticity, whereas others increase their gait asymmetry with behavioral compensation strategies to improve gait.”

*20. (340) Ok, but is your solution the best compromise? How can you demonstrate it? See previous question about model parameters/architecture and, more broadly the question of whether VAE are better than other dimensionality reduction techniques…*
20. Answer:
A valid question. However, we think that this as one of the first exploratory studies might be the starting point of future studies to further explore the field of using alternative methods to analyze IMU or movement data in general. If it is the best and the current settings are optimal is outside the scope of this study. We integrated this comment in the discussion as a recommendation for future studies.

Reviewer #2: Overall, this is an interesting study that provides useful new information to researchers using the IMU technology in the field of stroke rehabilitation. I have a couple of major concerns related to the lack of explanation regarding a statistical analysis that the authors used and justification for 5-second epochs.

*1. The authors did not provide any explanation regarding the statistical analysis that they used in the manuscript. A detailed explanation of their statistical analysis is necessary to be explained in the manuscript.*
1. Answer:
Thank you for your suggestion. We have added information to the statistical analysis section to inform the reader why we chose to evaluate the reliability, differences between healthy and stroke and the progression.
1. Textual change (lines 450-474):
“2.6 Statistical Analysis
The statistical analysis consisted of three parts. In the first part, the between-day test-retest reliability of the latent feature scores were calculated to indicate if the latent features are consistent. In the second part, the differences in latent feature scores between healthy and individuals after stroke were calculated to determine if the latent features capture information that differs between healthy and stroke. In the third part, the changes in the latent feature scores over time during rehabilitation were determined to indicate if the latent features are responsive to rehabilitation.”

*2. The data were segmented into 5-second epochs. Why was this segmentation necessary and why was 5-second? The justification for these aspects is necessary to be included in the manuscript.*
2. Answer:

The 2MWT measurements were segmented into 5-seconds for several reasons. Firstly, a Variational AutoEncoder requires a considerable amount of data to train. Therefore, by splitting the data into subsections we increased the amount of input data. Secondly, a 5-second measurement during a 2MWT presumably already contains relevant information regarding an individual’s gait pattern. For example, in 5 seconds, the IMU will capture 4/5 strides of faster individuals, whereas the data of slower individuals might only include 2 strides. Thirdly, a longer measurement duration is more difficult for the model to accurately reconstruct, since the amount of information and noise increases. Therefore, to achieve a similar reconstruction error, more latent features are required. Lastly, we aimed to segment the 2MWT data to calculate average scores for latent features, as averaging these values reduces the likelihood of outliers influencing the analysis.

Future research should further investigate the model architecture and parameters, including data length, to evaluate if better results can be achieved.
2. Textual change (lines 334-339):
“Fourth, spanning from the second to second-to-last stride, all 2MWT were split up into epochs of 512 samples, starting in a stance phase, with approximately 50% overlap. Data were split up into segments with 50% overlap to increase the amount of data for the VAE to learn, as a considerable amount of data is required to accurately estimate the model coefficients. An epoch length of 512 samples (5.12 seconds) was chosen to encompass multiple strides per epoch, thus capture relevant information about an individual’s gait pattern.

*3. Lines 99-101: The way you numbered seems confusing. Maybe another way looks better such as 1), 2), 3) or a), b), c).*
3. Answer:
Thank you for your suggestion. We have changed the numbers to the suggested format.
3. Textual change (lines 246-250):

“Moreover, we aimed to investigate the relevance of the latent features by determining 1) the reliability of the latent feature scores; 2) if the latent feature scores can be used to discriminate between people after stroke and a control group; 3) and lastly, if the latent feature scores can be used to map gait recovery, providing more information than just gait speed, in clinical stroke rehabilitation.”
 *4. Line 281: Explanation of abbreviations (L1-8) needs to be added to Table 2 legend.*

4. Answer:
4. Textual change (lines 629):

|  | Test-Retest (M = 55) | |  | Stroke (M = 68) |  | Healthy (M = 42) |  | Differences | | |
| --- | --- | --- | --- | --- | --- | --- | --- | --- | --- | --- |
|  | ICC [CI] | MDC (SEM) |  | *µ*(*σ*)[min*,* max] |  | *µ*(*σ*)[min*,* max] |  | t-statistic | p-value | Effect size |
| L0* | 0.75 [0.61,0.85] | 2.214 (0.799) |  | -0.3(0.4)[-1.2, 1.0] |  | 1.0(1.6)[-1.4, 3.4] |  | 5.3 | *<*0.01 | 1.24 |
| L1* | 0.847 [0.75,0.91] | 1.326 (0.478) |  | 0.1(0.6)[-2.3, 1.2] |  | -1.7(0.6)[-2.7, 0.0] |  | -14.2 | *<*0.01 | 3.00 |
| L2* | 0.851 [0.76,0.91] | 1.126 (0.406) |  | 0.0(0.6)[-1.9, 1.3] |  | -0.1(0.8)[-1.7, 1.4] |  | -0.9 | 0.356 | 0.15 |
| L3 | 0.481 [0.25,0.66] | 1.821 (0.657) |  | -0.0(0.4)[-0.8, 0.9] |  | 0.2(0.4)[-0.5, 1.4] |  | 2.0 | 0.052 | 0.50 |
| L4 | 0.452 [0.21,0.64] | 2.108 (0.761) |  | -0.0(0.4)[-1.6, 0.6] |  | 0.4(0.5)[-1.0, 1.3] |  | 3.6 | *<*0.01 | 0.91 |
| L5* | 0.899 [0.83,0.94] | 0.831 (0.3) |  | -0.4(0.4)[-1.3, 0.3] |  | -1.6(0.9)[-2.9, 0.9] |  | -7.9 | *<*0.01 | 1.87 |
| L6 | 0.618 [0.42,0.76] | 1.773 (0.64) |  | 0.2(0.5)[-1.3, 1.4] |  | -0.5(0.6)[-1.5, 0.9] |  | -5.8 | *<*0.01 | 1.30 |
| L7* | 0.796 [0.67,0.88] | 1.426 (0.514) |  | -0.2(0.7)[-1.0, 2.1] |  | -0.5(0.9)[-1.5, 1.6] |  | -1.8 | 0.072 | 0.38 |
| L8 | 0.684 [0.51,0.8] | 1.197 (0.432) |  | -0.4(0.6)[-1.7, 0.8] |  | 0.0(0.3)[-0.5, 0.6] |  | 5.5 | *<*0.01 | 0.79 |
| L9 | 0.431 [0.19,0.62] | 2.138 (0.771) |  | -0.1(0.3)[-1.0, 1.0] |  | -0.3(0.5)[-1.5, 0.8] |  | -1.6 | 0.108 | 0.51 |
| L10 | 0.369 [0.11,0.58] | 2.05 (0.74) |  | 0.1(0.4)[-0.9, 1.4] |  | 0.2(0.4)[-0.5, 1.4] |  | 1.5 | 0.132 | 0.25 |
| L11 | 0.704 [0.52,0.82] | 1.208 (0.436) |  | -0.1(0.4)[-1.0, 0.7] |  | 0.5(0.5)[-0.4, 1.6] |  | 6.2 | *<*0.01 | 1.36 |
| Gait speed* [m/s] | 0.963 [0.92, 0.98] | 0.137 (0.049) |  | 0.4(0.3)[0.1, 1.1] |  | 1.1(0.2)[0.6, 1.4] |  | 15.2 | *<*0.01 | 2.6 |
| Variables with a good to excellent reliability are marked with a *.  Abbreviations: M = measurements; m = meters; s = seconds; L = Latent Feature. | | | | | | | | | | |

*5. Some figures missed explanations of abbreviations in their legend. Please double-check and add explanations if needed.*

5. Answer:
Thank you for your attentiveness. We have made several changes to the figure descriptions.
5. Textual change (lines 582-590; 615-621; 660-671):

“Figure 3: Example of an original signal and its reconstructed version after being processed by a VAE. The original signal is a 512 X 6 epoch that consists of a 5.12 seconds (s) measurement with triaxial acceleration (Ax, Ay, Az) and angular velocity (Gx, Gy, Gz) data. The upper panel displays the normalized original and reconstructed acceleration. The lower panel shows the normalized original and reconstructed angular velocity. The reconstructed signal has a strong resemblance to the original signal, as indicated by visual inspection. Overall, this image demonstrates the effectiveness of using a VAE to reduce the dimensionality of a complex signal while maintaining its important features, such as the distinct strides visible in the original signal. More examples are available via: [edu.nl/p3kv4](https://edu.nl/p3kv4).”

“Figure 4: Normalized distribution of the reconstruction error as the Mean Squared Error (MSE) of gait data of people after stroke (blue) and healthy controls (orange). The majority of the epochs from people after stroke were reconstructed with an error below 0.004, while the average reconstruction error of the healthy control group was substantially larger. This indicates that the VAE was less accurate in the data-reduction and reconstruction of the data of the healthy controls.

“Figure 5: Distribution of the latent features (L0-L11) and gait speed (z-normalised). The results of the healthy participants are colored in orange, the results for people after stroke are colored in blue. The * indicates a variable with a high-excellent reliability. The # indicates a significant difference between healthy participants and people after stroke. The height of the distributions on the y-axis indicates the range of the latent variable. The width of the distribution on the x-axis indicates the height of the peak. Since the latent variables are computed with a VAE, the distributions of the stroke group are approximately normally distributed around 0. Visual inspection indicates some differences between the healthy and stroke group. First, for the healthy participants, L0 appears to follow a bi-modal distribution. Second, L1 demonstrates a peak at another height than the peak of the stroke group.”
